# Patient reported outcome measures for measuring dignity in palliative and end of life care: a scoping review

**DOI:** 10.1186/s12913-017-2450-6

**Published:** 2017-08-22

**Authors:** Bridget Johnston, Kate Flemming, Melanie Jay Narayanasamy, Carolyn Coole, Beth Hardy

**Affiliations:** 1 School of Medicine, Dentistry & Nursing, College of Medical, Veterinary & Life Sciences, University of Glasgow, 57-61 Oakfield Avenue, Glasgow, G12 8LL UK; 2 Department of Health Sciences, University of York, Heslington YO10 5DD, York, UK; 3 School of Health Sciences, University of Nottingham, Nottingham, NG7 2UH UK

**Keywords:** Patient reported outcome measures, Palliative care, End of life care, Dignity, Scoping review

## Abstract

**Background:**

Patient reported outcome measures are frequently used standard questionnaires or tools designed to collect information from patients regarding their health status and care. Their use enables accurate and relevant insight into changes in health, quality of life, and symptom severity to be acquired. The purpose of this scoping review was to identify PROMs that had been subject to rigorous development and were suitable for use in palliative and end of life care for clinical practice and/or research purposes. The review had a specific focus on measures which could be used to assess perceptions of dignity in these contexts.

**Methods:**

A scoping review of English-language papers published between 2005 and 2015. Searches were devised in conjunction with an information science specialist and were undertaken in Medline; PsycINFO; EMBASE; CINAHL; Social Science Citation Index; ASSIA; CENTRAL; CDSR; DARE; HTA; Oxford PROM Bibliography; PROQOLID, using dignity related terms such as personhood; dignity or dignified; patient-centred care; which were linked (via the Boolean operator “AND”) to care-related terms such as terminal care; hospice care; palliative care; end of life. Papers were assessed against inclusion criteria and appraised for quality.

**Results:**

The search strategy produced an initial 7845 articles. After three rounds of eligibility assessment, eight articles discussing eight patients reported outcome measures were found to meet the inclusion criteria and were included in the final review. These underwent a thorough critical appraisal process. All seven studies were empirical research focused on the development and testing of a PROM.

**Conclusions:**

The eight patient reported outcome measures had all undergone some psychometric testing, and covered dignity aspects suggesting that they could be considered for use for research purposes to assess dignity. There were also indications that some could be implemented into a clinical setting. However, each measure had limitations and scope for further development.

## Background

### Patient reported outcome measures

Patient reported outcome measures (PROMs) are standard questionnaires or tools used to acquire information from patients regarding perceptions of their wellbeing and functional status [1, 2]. They operate by recording changes over time, following an initial baseline measurement being taken [3]. These tools can be used in areas such as palliative care to provide a means of assessing and monitoring care [3]. Measuring outcomes in healthcare require patients to be the primary givers of information, in order to gain accurate and relevant insight into changes in health, quality of life, and symptom severity [3]. PROMs are increasingly encouraged for use in clinical practice, audit and research [3, 4]. In research, PROMs are pivotal for testing the effect of an intervention [4–6]. PROMs are advocated for their ability to elicit information directly from the patient, prioritise the perspective of the patient, and when sufficiently validated, they are able to deliver an accurate assessment of the clinical population [1, 3]. PROMs are the ideal strategy to measure patient-centredness by giving patients the opportunity to assess and convey the extent to which they feel that care received meets their values and needs [7]. Furthermore, PROMs can help highlight aspects of care that need to be improved [7]. In addition, assessments allowed through PROMs are crucial to care provision [8].

In palliative and end of life care, the use of PROMs is encouraged to allow palliative care interventions to be assessed, which ultimately provides guidance for teams working in this area. Furthermore, UK supportive and palliative care guidance recommends that systematic assessments are crucial to the provision of supportive care [8, 9]. It has been suggested that the growing landscape of research into experiences of dying have highlighted the importance of focusing on quality improvement in the area of end of life care [10].

The primary goal of palliative care should be to ensure high quality of life for patients who have advanced incurable disease. This involves attending to the person’s psychological, social, and spiritual needs, so ultimately the endeavour of palliative care is to generate the best quality of life for patients and their families [11]. Outcome measures should then strive to assess whether this has been achieved [11]. Hearn and Higginson [12] suggest that PROMs in palliative care should be developed to capture key goals of palliative care philosophies including improving quality of life before death, attending to symptoms, and providing support for loved ones. The information gained from outcome measures is useful since it produces a clinical picture of patients, helps improve symptom assessment, enhances communication between service users and staff, achieves better patient satisfaction, and ultimately supports the delivery of person-centred care [6, 13–15]. In turn, this can help improve the quality of the service [6], and supports shared decision making between patients and staff [15]. In addition, data elicited from PROM reporting can also generate key information that may not be otherwise routinely recorded or available in medical and nursing records [14].

### Challenges to using PROMs in palliative care

The palliative care environment can pose challenges to the effective use of PROMs [12]. For example, the patient with palliative care needs may be unable to complete PROMs due to advanced illness or cognitive impairment. This potentially leads to situations where PROMs are only used for patients with less problems and therefore excludes those with more severe issues. This also compromises the validity of the PROM, since the use of proxy individuals may not reflect the true perceptions of the patient [16]. Collins et al. [6] propose the use of the term “person-centred outcome measures”, to reflect situations where someone other than the patient (such as a family member of healthcare professional) fills in the measure, but still strives to capture the patient’s priorities. In their systematic review on the use of outcome measures in palliative care, Hearn and Higginson [12] found that no outcome measure in their final included selection was designed to cover more than one relevant domain in palliative care. This is echoed by a more recent review [10] focusing on end of life care, in which they identify a need to take into account *“the full spectrum of patients’ and caregivers’ end-of-life experience”* (2007:1849). In addition, measuring the diverse outcomes of palliative care requires a holistic approach that includes consideration of what may be seen as obscure domains such as psychosocial and spiritual dimensions [3]. These authors also propose that the realm of palliative care is increasingly complex because of the spectrum of needs and conditions that are present, which then makes measurement difficult. Moreover, certain outcomes, such as quality of life, may be difficult to measure, since this is a multidimensional and subjective concept [7].

There may also be challenges to both the selection of PROMs and implementing them in clinical practice, due to lack of time, resources and training [1]. The unique needs of patients requiring palliative care means that outcome measurement can be challenging [3]. In addition, ethical concerns arise around issues such as whether measuring symptoms might intrude on the patient’s preferred use of time at the end of life; whether it is appropriate to measure symptoms that may be complex and inter-related in presentation; [3]. It is also highlighted that [13] the possible drawbacks of using paper-based outcome measures, suggesting that they may not be flexible; are limited in terms of language and literacy requirement; may not be appropriate for individuals from minority groups; and the inability to generate instant clinical information. Similarly others question the usefulness of questionnaire content, by suggesting that they do not offer clear distinction between statistical and clinical significances [16]. However, in cancer settings, [12] PROMs are useful in helping to distinguish between physical, emotional, and social problems and also providing a means to monitor effects.

### The ideal attributes of PROMs

A substantial body of work has been undertaken which provides guidance and recommendations for the development, selection, implementation and use of PROMs in palliative and end of life care, in addition to, addressing some of the identified challenges associated with the use of PROMS. Ideally, PROMs in end of life care should aim to cover the key areas of hope, spirituality, symptom control, self-concept, the therapeutic consultation, and dignity [12]. In order to enhance the evidence base for end of life care, PROMs should capture patient and caregiver experiences and undergo reliability and validity testing [10]. As well as, being selected and implemented from a reliable evidence base [4] Specific projects have prioritised developing and providing guidance for PROMs in palliative and end of life care. These are summarised in Table 1.

**Table 1:** Recommendations for PROMs from key projects

Project	Summary	Key recommendations
Methods of Researching End of life Care (MORECare)- Higginson et al. (2013)	Dedicated to producing evidence-based guidance on methods to help in the design and conduct of research in end of life care. Produced a statement/checklist of key points	Outcome me:asures should be: 1. Short 2. Responsive to change over time 3. Used for both clinical practice and research 4. Have validity and reliability in the relevant population 5. Able to capture clinically important data 6. Easy to administer 7. Easy to interpret 8. Applicable across different care settings
Reflecting the Positive DiveRsities of European Priorities for ReSearch and Measurement in End-of-Life Care- Bausewein et al. (2011)	PRISMA focuses on bringing about best practice and supporting research and outcome measurement in end of life care across Europe. Booklet produced to offer support and guidance for understanding, selecting, and using PROMs in palliative care	Outcome measures should be: 1. Valid: *Face and content validity; Criterion and construct validity* 2. Reliable: *Inter-rater reliability; Test-retest reliability; Internal consistency* 3. Appropriate and acceptable for clinical use 4. Responsive to change/ able to detect changes 5. Interpretable/ translatable to meaningful information 6. Translatable to other languages
Outcome Assessment and Complexity Collaborative (OACC) Suite of Measures- Murtagh et al. (2014)	Working to monitor the implementation of outcome measures into routine clinical practice. Developed a suite of recommended measures for palliative care and guidance on implementation.	Outcome measures should be: 1. Reflective of the key domains of palliative care e.g. stage of illness; patient’s functioning; symptoms; other key concerns; impact on patient’s and family’s quality of life

As part of the Methods of Researching End of life Care (MORECare project) [17] a checklist of components was generated to help researchers in designing and conducting intervention studies in end of life care. This work provides specific recommendations for the design of outcome measures and proposes guidelines for format aspects including short lengths; ease in administering and interpreting; and ensuring that the tool is adaptable to different settings. In addition, this body of work emphasises the importance of the tool being validated and tested for reliability. Similarly, the PRISMA project [18] highlights the importance of validity and reliability and proposes what types of validity and reliability testing should be done. In addition, this work recommends that outcome measures have the ability to be translated into other languages. Both MORECare and PRISMA state that responsiveness to change over time is also a priority for outcome measures to demonstrate [3, 12]. A further key body of work is Witt et al.’s Outcome Assessment and Complexity Collaborative (OACC) Suite of Measures [19], which emphasises the need for outcome measures to capture the domains relevant to palliative care. These recommendations were taken into account when formulating the critical appraisal tool for this current scoping review (Please see section *Critical appraisal of PROMs* under “Methods”, for further detail).

However, as well as meeting particular quality standards, PROMs also have a responsibility to measure phenomena that are significant for, and prioritised by patients [19]. Dignity is recognised as being integral to human rights and a priority for recipients of palliative and end of life care [20, 21]. Dignity is defined as having a quality that is deemed worthy of receiving respect and, in turn, also promotes self-respect [22]. The tenets of dignity, which revolve around kindness, humanity, and respect, are arguably neglected by professionals working under challenging conditions, including time pressure [23]. Attention to conserving dignity in palliative and end of life care settings is welcomed by patients and the research landscape is increasingly dedicated to developing clinically-appropriate interventions which fulfil this aim [21, 24–26]. Nevertheless, dignity is not always made a priority when developing PROMs [27]. Therefore, PROMs which extract dignity-related outcomes are important in order to assess the extent to which patients are satisfied that their dignity is being conserved by care-giving activities and approaches.

### Previous reviews

This current review intended to identify key PROMs used in palliative and/or end of life care that related to dignity and were related to and relevant for the nursing context, i.e. care delivered by nurses as part of a multi-professional team caring for people with palliative care needs in the last months of life. In addition, this review, uniquely, seeks to find PROMs which specifically help to measure patient perceptions of dignity, a focus which has not been exclusively attended to by previous reviews. Moreover, as evidence-based practice is central to both medical and nursing care environments [28–31], it was vital that a review of available evidence was undertaken to ensure that appropriate outcome measures are being used in research and clinical practice.

An early review [3] sought to identify outcome measures which enable palliative care interventions used for patients with advanced cancer to be evaluated. Forty-one measures made it into the final review, including the Edmonton Symptom Assessment Schedule (ESAS), and the McGill Quality of Life Questionnaire (MQQL). These authors proposed key criteria to use when assessing measures, which revolve around validity and reliability considerations, as well as responsiveness to change. The final seven included measures were found to target physical, psychological and spiritual domains to some extent, but no one measure fulfilled all the requirements of an ideal tool. Hearn and Higginson [12] questioned whether such an ideal tool can be achieved, but also stand firm that this endeavour should not be neglected. Mularski et al.’s [10] review had similar aims in seeking to identify appropriate end of life measures, which had undergone good psychometric testing. A larger number of measures were found with 95 being identified from the review; thirty-five from a previous systematic review; and finally 64, which measured end of life experience. The measures varied in the focus, and revolved around quality of life; physical, emotional, spiritual areas; advanced care planning; and caregiver wellbeing. The review highlighted the lack of robust testing for most measures, and a significant gap in appropriate measures to address continuity of care, advanced care planning and spiritual issues. However, the authors acknowledge that amongst the limitations of their review, their broad definition of “end of life” risks being both over-inclusive and possibly not inclusive enough.

Other reviews chose specific measures to focus on; Collins et al. [6], aimed to appraise evidence for use of the Support Team Assessment Schedule (STAS) and the Palliative care/ Patient Outcome Scale (POS). The review highlighted a clear increase in use of both measures in diverse settings and countries, with both being accepted as validated and reliable tools used to assess symptoms and needs. In addition, as well as being part of clinical assessments, the tools were also used in the evaluation of interventions. The POS remained more popular than the STAS, and the authors propose that this may be because the POS is inherently patient-focused by being a PROM. However, the review risks having an element of bias, since their focus on these two measures could arguably have been influenced by the fact that most of the authors had involvement in the development of them.

Finally, Parker and Hodgkinson [3] were interested in determining the reliability, validity, and feasibility of outcome measures used specifically in long term care facilities. Motivated by the lack of work previously addressing the area of long term facilities, the review identified ten outcome measures appropriate for this setting, with the Family Perceptions of end-of-life Care Scale (FPCS) being deemed most appropriate, based on the rigorous development and testing it has undergone. However, the authors found that some validity aspects were difficult to determine, thus compromising this aspect of appraisal.

Development of PROMs research is in line with recent health policy recommendations [9, 32, 33], and acknowledges the importance of prioritising the patient’s perspective, thus supporting patient-centred care. This current scoping review is both relevant and timely, in particular, because no recent reviews focus exclusively on PROMs within the nursing context nor with a focus on dignity. Undertaking a scoping review, as opposed to any other form of knowledge appraisal, allowed us to specifically address an exploratory research question which aimed to map key types of evidence as well as identifying any gaps in that evidence [34]. Moreover, this review intends to identify high quality PROMs that can potentially be used to confidently evaluate palliative and/or end of life interventions, aligning with the MRC’s recommendations [35] for evaluating complex interventions. This will strengthen the evidence-base of these interventions and support their implementation to practice.

## Methods

### Objectives

To map the evidence and quality of PROMs which assess dignity, and have been used for patients in palliative and/or end of life settings. The key question guiding this scoping review was as follows:

What are the key PROMs available that are used to measure dignity in palliative and end of life care for clinical practice and/or research purposes?

As well as measuring dignity, PROMs were expected to show high standards of development, evidenced by their ability to meet key critical appraisal requirements (see Table 5).

Inclusion criteria were developed early on to guide the narrowing down process of retrieved articles. This conveyed the key criteria that papers were expected to achieve in order to be considered for inclusion. The inclusion criteria are displayed in Table 2 and were used in Stages 1 and 2 of the narrowing down process (please see section *Study selection*).

**Table 2:** Inclusion criteria

Review inclusion criteria	Clarification/Justification
1. Papers must: - Report an outcome measure that has been subject to validity and reliability testing - Be a systematic review of outcome measures	o Interested in papers that that describe the development and testing of outcome measures that have been through reliability and validity testing or are systematic reviews of existing measures
2. Outcome measure must be relevant to any patient experiencing an illness or condition for which they are receiving palliative or end of life care	o *Palliative care*- An approach aiming to improve the quality of life of patients who are facing life-threatening illness, through the prevention, assessment and treatment of pain and other physical, psychosocial and spiritual problems. Not intended to hasten or postpone death (World Health Organization 2012) o *End of life care-* Care that helps those with advanced, progressive, incurable conditions; adults who may die within 12 months; and those with life-threatening acute conditions. It also covers support for the families and carers of people in these groups. o Outcome measure –is: A patient reported outcome (PRO) is any direct patient report about a health condition or its treatment. A PROM is a questionnaire, or series of questions, that ask patients to assess their views on their health, or the impact of received healthcare on their health. PROMs have been widely used across a range of research settings, and more recently in clinical practice. PROMs may be completed as pen and paper questionnaires, or as new technologies allow via computer or mobile formats. Two broad categories of PROM are described: generic and disease- or condition- specific. o Generic measures are not age-, disease-, or treatment-specific. They ask about multiple aspects of overall health and quality of life that have relevance to patients with different conditions and the general population. o Specific measures may be specific to a particular disease or a patient population (, or a specific aspect of health o We are looking for specific measures for palliative and or end of life care and *ideally* not disease or condition specific
3. Target patient population must include people receiving palliative and/or end of life care/care of the dying	o See definition of *Palliative care* and *end of life care* for point 2.
4. Papers must include indication that one or more relevant palliative and/or end of life domains (physical, psychological, social and spiritual) have been measured	Based on World Health Organisation definition o *Physical-* addressing pain and other distressing symptoms present in the patient o *Psychological-* addressing psychological (mental and/or emotional) aspects of the patient o *Social-* Offering a support system to help patients live as actively as possible until death; enhancing quality of life for patients o *Spiritual-* Addressing spiritual (meaningful activity, personal growth, connections) aspects of the patient
5. Papers must be written in English-language	We do not have the resources or time to translate measures
6. Papers must have been published between 2005 and 2015	We are interested in papers published within the last 10 years, since early scoping identified that this period was more likely to produce relevant papers/ discussion of key patient reported outcome measures
7. Papers must be focused on populations over 18 years of age	We are not looking for measures that re chid or adolescent specific

### Scoping review method

The methods for scoping reviews have come under recent scrutiny [34] and have been developed from the early work of Arksey & O′ Malley [36] with an aim of producing consistent use of methodological guidance and reporting. We followed five of the six methodological steps outlined in the Arksey & O’Malley [36] and Levac et al. [37] framework recommended by Colquhoun et al. [34]. The final stage ‘Consultation’ is optional and involves opportunities for consumer and stakeholder involvement which were beyond the scope of this review:

1. Identifying the research questions
2. Identifying relevant studies
3. Study selection
4. Charting the data
5. Collating summarising and reporting the results

### Identifying relevant studies

**Fig. 1:**
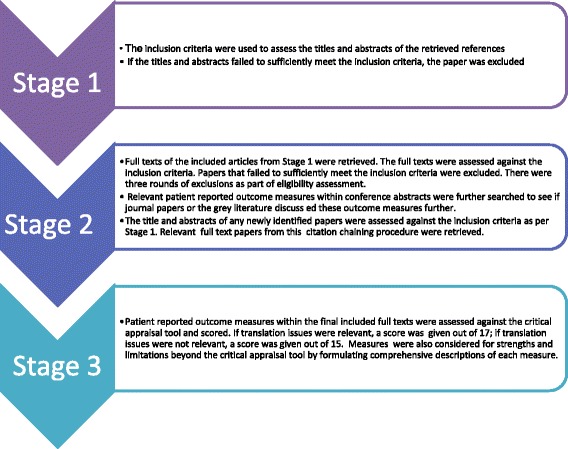
The stages of narrowing down texts (Prisma diagram)

Figure 1 provides an overview of the key stages of the review process that we undertook.

The search strategy was developed through collaborations between the research team (including a senior clinical academic with expertise in palliative and supportive care; a senior academic with expertise in evidence synthesis; a lecturer in adult nursing with clinical and research experience in community nursing), and an information scientist. In addition to consultations with the expert in evidence synthesis and information scientist, keywords and free text terms were also informed by an early scoping exercise, from which literature was scrutinised for relevant terminology and synonyms. The Medical Subject Headings (MeSH) term browser, provided by the United States’ National Library of Medicine, was also used to identify appropriate index terms. The final search strategy consisted of dignity related terms such as personhood; dignity or dignified; patient-centred care; which were linked (via the Boolean operator “AND”) to care-related terms such as terminal care; hospice care; palliative care; end of life. A sample search strategy from the MEDLINE database is displayed in Table 3.

**Table 3:** MEDLINE search strategy

	Search term	Number of hits
1.	personhood/	3271
2.	humanism/	2998
3.	self concept/	47,018
4.	(dignity or dignified). ti,ab.	5091
5.	(personhood o person-hood). ti, ab.	791
6.	(self-worth or self-concept or self-esteem). ti, ab.	18,619
7.	patient-centred care/	12,034
8.	(person adj (centred or centered or focused)).ti, ab.	1781
9.	(patient adj (centred or centered or focused)).ti. ab	11,059
10.	(client adj (centred or centered or focused)).ti, ab.	1108
11.	(user adj (centred or centered or focused)).ti, ab.	526
12.	((whole person or holistic) adj2 (need$ or care or caring)).ti, ab.	1781
13.	or/1–12 (88873)	88,873
14.	Terminal Care/	22,110
15.	Hospice Care/	4852
16.	Palliative Care/	42,211
17.	“Hospice and Palliative Care Nursing”/	131
18.	Hospices/	4440
19.	Palliative Medicine/	35
20.	Terminally Ill/	5562
21.	end of life.ti,ab.	13,365
22.	(end-stage$ or endstage$).ti, ab.	48,621
23.	(life threatening or life limiting).ti, ab.	59,165
24.	((final or last) adj3 days).ti, ab.	10,417
25.	(terminal$ adj3 (ill$ or stage$ or phase or prognosis or disease$ or cancer$)).ti, ab.	14,027
26.	(terminal$ adj3 (care or caring or therap$ or treatment$ or intervention$)).ti, ab.	3908
27.	(terminal$ adj2 patient$).ti, ab	5078
28.	palliat$.ti, ab.	52,801
29.	hospice$.ti, ab.	8971
30.	dying.ti, ab.	26,751
31.	or/14–30	241,674
32.	13 and 31	3374
33.	(death adj2 (dignity or dignified)).ti, ab.	596
34.	32 or 33	3585

Searches were conducted within key nursing, medical, psychological, and social sciences databases to identify papers from the period of 2005–2015. This period was deemed appropriate, since a preliminary exercise and consultation with the research team indicated that relevant papers, featuring the most advanced dignity-related PROMs, were most likely to emerge within this ten-year timescale. The databases that were consulted were ASSIA; CINAHL; Cochrane Central Register of Controlled Trials (CENTRAL); Cochrane Database of Systematic Reviews (CDSR); Database of Abstracts of Reviews of Effects (DARE); EMBASE; Health Technology Assessment Database (HTA); MEDLINE; Oxford PROM Bibliography; PROQOLD; PsycINFO; and Social Science Citation Index.

In addition, grey literature searching was carried out using Google search engine, grey literature databases, and relevant charity and organisation websites. Databases consulted were the Bielefeld Academic Search Engine (BASE) and OpenGrey. Organisation and charity websites, which included Royal College of Nursing, Age UK and Department of Health were consulted. Grey literature searching was supported by consultations with experts in the field. Key terms were adapted from the main search strategy. The grey literature failed to provide any appropriate PROMs which stood up against the criteria outlined in the critical appraisal tool.

### Study selection

**Fig. 2:**
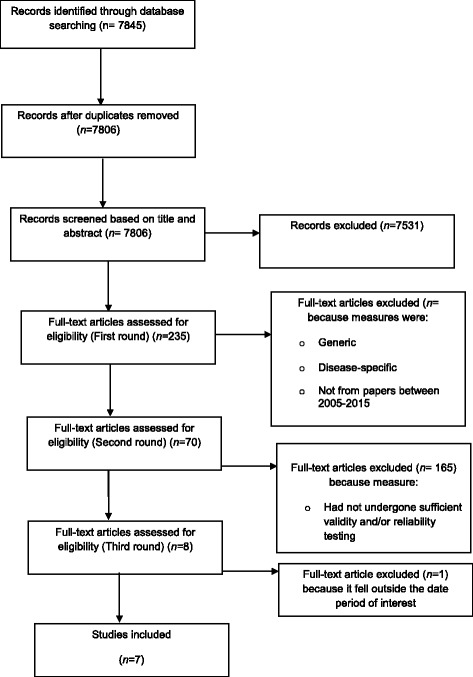
PRISMA flow diagram

Database searches retrieved 7845 results which were exported to a referencing management program (Endnote ×6). The numbers of retrieved articles from each database are displayed in Table 4. Thirty-nine duplicate papers were identified and removed, leaving a total of 7806. A narrowing down process was established which consisted of systematic stages. Stage 1 involved members of the research team (MN, BJ, CC) assessing title and abstract against the inclusion criteria. If the titles and abstracts failed to meet the inclusion criteria, the paper was excluded. In Stage 2, the full texts were assessed against the inclusion criteria (BJ; CC; KF; BH; MN). Papers which failed to meet the inclusion criteria were excluded. There were three rounds of eligibility assessments for full texts papers, before the final seven papers were agreed. The narrowing down process is captured in a PRISMA flow diagram as conveyed in Fig. 2.

**Table 4:** Numbers of articles retrieved from databases

Database	Results	After deduplication
MEDLINE & MEDLINE In-Process	3146	3065
EMBASE	4530	2073
PsycInfo	1832	1167
CINAHL	3081	939
Social Science Citation Index	1382	476
ASSIA	440	88
Cochrane Central Register of Controlled Trials (CENTRAL)	101	11
Cochrane Database of Systematic Reviews (CDSR)	4	3
Database of Abstracts of Reviews of Effects (DARE)	25	11
Health Technology Assessment Database (HTA)	4	4
Oxford PROM Bibliography	21	4
PROQOLID	4	4
Total	14,570	7845

### Critical appraisal of PROMs

Critical appraisal of the PROMS was undertaken as part of the scoping review as recommended by Daudt et al. [38]. The critical appraisal guidance was developed based on previous work in the field [3, 12, 13, 39] and consisted of key qualities that the patient reported outcome measures being discussed in the papers were expected to have. In line with the recommendations and guidance offered by MORECare, PRISMA, and OACC projects (please see Table 1), the critical appraisal tool of this current review takes into account aspects of formatting; validity and reliability testing; clinical responsiveness; translation expectations; and other issues deemed relevant when appraising PROMs in palliative and end of life care. This tool is displayed in Table 5, and was used in stage 3 of the narrowing down process. This consisted of members of the research team (MN; BJ) assessing the PROMs identified in the papers from stages one and two, against the critical appraisal tool.

**Table 5:** Critical appraisal tool

Screening question	Responses and key prompt questions to help make the decision
1. FORMAT Is the measure relevant for use in palliative^1^ and/or end of life care^2^?	o Yes [] o Can’t tell [] o No [] ➢ *Has the measure been developed for use for people with conditions that require palliative and/or end of life care?* E.g. *including but not limited to cancer, neurological conditions, heart failure, chronic obstructive pulmonary disease* ^1^Palliative care is understood as an approach aiming to improve the quality of life of patients who are facing life-threatening illness, through the prevention, assessment and treatment of pain and other physical, psychosocial and spiritual problems. It is not intended to hasten or postpone death (World Health Organization, 2012). ^2^ End of life care is care that helps those with advanced, progressive, incurable conditions; adults who may die within 12 months; and those with life-threatening acute condition. It also covers support for the families and carers of people in these groups (NICE, 2013)
Is the measure administratively manageable?	o Yes [] o Can’t tell [] o No [] ➢ *Is the measure freely available?* ➢ *Is the measure easy to access?* ➢ *Is the measure easy to follow, use and understand?* ➢ *Can the measure be completed within a short time frame? (max 15 mins)-check* ➢ *Is there adequate guidance over how scores should be interpreted?* ➢ *Can the measure be used in clinical practice?* ➢ *Will it fit into clinical routines?*
Is the measure short?	o Yes [] o Can’t tell [] o No [] ➢ *Is the outcome measure no more than 4 pages of A4 paper?*
2. DATA COLLECTION TIME POINTS Does the measure have a clear baseline and subsequent clear time points for measures to be taken?	o Yes [] o Can’t tell [] o No [] ➢ *Is there clarity about when the measure should first be used with patients?* ➢ *Is there clarity about when the measure should be used after this first time?* ➢ *Is there clarity about how many times the measure should be used with patients?*
3. VALIDITY TESTING Has the measure been tested for validity?	o Yes* [] o Can’t tell [] o No [] *If Yes: Is there evidence of content validity^3^? o Yes [] o Can’t tell [] o No [] Is there evidence of criterion validity^4^? o Yes [] o Can’t tell [] o No [] Is there evidence of construct validity^5^? o Yes [] o Can’t tell [] o No [] ➢ ^*3*^ *Content validity: Is it clear what concept is being measured? Does the measure include all items that are relevant to the concept being measured?* ➢ ^*4*^ *Criterion validity: Does the measure correlate with superior measures, considered as a “gold standard” tests?* ➢ ^*5*^ *Construct validity: Does it measure the underlying concept of interest?* *NB. Depending on what the concept being measured is, you need to look this up to determine whether the items within the measure are adequately representing the overall concept.*
4 RELIABILITY TESTING Has the measure been tested for reliability	o Yes* [] o Can’t tell [] o No [] *If Yes: Is there evidence of test-retest reliability?^6^ o Yes [] o Can’t tell [] o No [] Is there evidence of internal consistency?^7^ o Yes [] o Can’t tell [] o No [] Is there evidence of inter-rater reliability?^8^ o Yes [] o Can’t tell [] o No [] ➢ ^6^ *Test-retest reliability: is there consistency in test results when administered on different occasions?* ➢ ^7^ *Internal consistency: do all items in the measure address the same underlying concept?* ➢ ^8^ *Inter-rater reliability: does the measure produce similar results when used by different observers?*
5 CLINICAL RESPONSIVENESS Is the measure able to detect clinically significant changes that take place over time?	o Yes [] o Can’t tell [] o No [] ➢ *Is the measure able to pick up on changes such as changes in perceptions of care, satisfaction with care, worries about care (if designed to do so). These indications may be given as scores.* ➢ *Is the measure able to acquire a score rating from the patient before an intervention is given to them? (if designed to do so)* ➢ *Is the measure able to acquire a score rating from the patient on at least one occasion after an intervention is given to them? (if designed to do so).* ➢ *Does the measure indicate what is counted as a clinically significant score change?*
6 ACCEPTABILITY AND APPLICABILITY Do the intended population find the measure acceptable to use?	o Yes [] o Can’t tell [] o No [] ➢ *Is the evidence that the participants on which the measure was used, have been asked to indicate whether they accept the outcome measure?* E.g. *giving them a questionnaire to find out what they think about the measure and/or conducting an interview with them to acquire their opinions*
Is the measure applicable to the clinical setting?	o Yes [] o Can’t tell [] o No [] ➢ *Is there evidence that the measure has been implemented in clinical settings?* ➢ *Is there a clear explanation as to how the measure has been or can be implemented in clinical settings?* ➢ *Is there evidence that healthcare professionals accept the measure?*
7. TRANSLATION *Only refer to this if author has indicated that translation of the measure occurred*	Has the measure been sufficiently translated? o Yes [] o Can’t tell [] o No [] ➢ *Have all items of the measure been translated?* ➢ *If not all items have been translated, is this justified?* Have the meaning behind concepts been sufficiently translated? o Yes [] o Can’t tell [] o No [] ➢ *Does the translated measure take into account the context/ culture relevant to the language it is being translated into?* ➢ *Are steps taken to ensure that meanings behind concepts are relevant to the people who will be using the measure in its new language?* ➢ *Has the translated measure been tested for validity, reliability, clinical responsiveness, acceptability and clinical applicability (see criteria 3,4,5,6).*

The specially developed critical appraisal tool comprises of seven key screening questions, with 17 specific areas to be scored. Where necessary, bullet pointed guidance is given under areas where it was anticipated that further clarification would be needed. The PROMs were scored based on the ability to answer “yes” to the relevant areas. For PROMs where translation was not relevant, there were 15 potential areas to score “yes” on (three under FORMAT; one under DATA COLLECTION TIME POINTS; four under VALIDITY TESTING; four under RELIABILITY TESTING; one under CLINICAL RESPONSIVENESS; and two under ACCEPTABILITY AND APPLICABILITY. Where considerations for translation were relevant, there were 17 areas to score “yes” on (15 as before with the additional two areas that come under TRANSLATION). In addition to using the tool, descriptive summaries of each PROM was produced to ensure that strengths and limitations not captured by the tool were identified.

### Charting the data

Table 6 provides a means of charting the data and is an overview of the final included articles. The table headings which assisted the charting process are informed by data extraction techniques employed by previous researchers undertaken similar work [3, 10]. Charting the data around PROMs has allowed useful summaries to be produced, which served to compliment the critical appraisal performed using the tool.

**Table 6:** measures included in final review

Measure	Key attributes	Strengths	Weaknesses	Critical appraisal SCORE
*1.The Palliative Nursing Quality Measure* ***:*** Cameron and Johnston [40]	Innovative; based on its unique foci on the key characteristics of the specialist nurse working in palliative care. This has not been an area attended to by other existing PROMs. Face and content validity were strengthened by input from the expert panels. Findings from the advisory group and panel feedback phases also suggest that the questionnaire is relevant to measure the quality of palliative care as provided by a specialist nurse, is administratively manageable, and appropriate for patients with palliative care needs, even when frailty is advanced.	A strength of this measure is that it does accommodate some free text input from participants, which means that a larger level of detail can be collected*.*	Only reports on face and content validity which partly accounted for it being the lowest-scoring measure based on our tool’s critical appraisal at Further testing around other validity aspects, such as criterion and construct are needed. In addition, reliability testing was also absent.	53.3% (8 out of 15).
2. Patient Dignity Inventory (PDI) Chochinov et a [41]	This is a 25-item tool, which is designed to assess dignity-related distress amongst people with end of life care needs. The PDI items were developed from the themes and subthemes encapsulated within Chochinov and colleagues’ Dignity Mode PDI as a self-report instrument that can be completed with assistance if necessary, that addresses appropriate issues across the physical, psychosocial, existential, and spiritual aspects of the patient’s experience. The study described in the current article explores the psychometric testing of the 25-item PDI amongst Canadian and Australian patient participants with end of life care needs, across three sites (*n* = 190). Various areas of psychometric testing were carried out.	The authors highlight that the PDI demonstrates strong face validity, and is adaptable to a range of care settings, such as community based locations as well as palliative care hospital units	The limitations addressed by the authors include the fact that the PDI should be robustly researched amongst younger patient populations and those with non-cancer conditions, since this current study consisted of largely older people with cancer illnesses.	93.3%/ (14 out of 15)
*3. Quality of Communication Questionnaire Assessing Communication about End-of-Life Care* Engleberg et al.’s [47]	The QOC instrument measures patients’ perspectives regarding satisfaction with health professionals communication (there are separate questionnaires for physicians and nurses) during end of life care. It was originally a four –item questionnaire [51, 52], but was extended to 17 due to considerations of ceiling effects.	One of only measures to specifically measure health care communication	Family reported data did not attain statistical significance as part of cross-respondent validation further testing is required. Sample selectivity techniques meant that participants were drawn from a subset of possible participants, and therefore it is not clear whether findings are applicable to those who did not participate, as well as, the wider population. The samples only covered two end of life care arenas: people receiving hospice care and people with COPD, which may restrict the appropriateness of the instrument to other people with end of life needs. Moreover, validity testing was not carried out on questionnaire items which were prospectively selected. Finally, the scales were not subject to some aspects of reliability testing, such as test-retest and responsiveness	66.7% (10 out of 15)
*4. Jacelon Attributed Dignity Scale* Jacelon and Choi [42]	This is an instrument dedicated to measuring attributed dignity amongst older adults in the community. The authors suggest that attributed dignity is a form of dignity, which involves ideas around self-value and perceived value from others. This concept of dignity was developed by Jacelon [53, 54] based on a study looking at older adults in hospital. The measure assesses the individual’s reflections on the attributed dignity that they did/did not experience in the previous week	Is a short measure using a consistent positive scoring approach, with higher scores equating to perceived greater attributed dignity	Further testing is required to establish whether the modified response format is feasible. In addition psychometric testing falls short of exploring inter-rater reliability, content validity, and criterion validity. Inability at this stage to be used clinically beyond research studies.	60.0% (9 out of 15)
*5. The Measurement Instrument for Dignity Amsterdam- for Long-Term Care facilities* Oosterveld-Vlug et al. [43]	The MIDAM-LTC is an instrument which assesses the extent to which aspects of a person’s life influences their sense of personal dignity	MIDAM-LTC enables dignity to be assessed more appropriately in long-term care settings, and for offering guidance to improve the dignity-conserving practice of caregivers. Modifying the measure to a 31 item tool was perceived by authors to improve feasibility whilst retaining comprehensiveness The MIDAM-LTC tool is unique by acknowledging that personal dignity is particularly vulnerable to being diminished in long-term care facilities, and therefore provides a useful means of assessing this amongst residents who are institutionalised.	Some key aspects of reliability testing were missing including internal consistency and inter-rater reliability	73.3% (11 out of 15).
*6. Problems and Needs in Palliative care questionnaire* Osse et al. [46]	Clinical tool that enables needs assessment in palliative care. The PNPC-sv is organised into different dimensions which are: Daily activities; Physical symptoms; Autonomy; Social issues; Psychological issues; Spiritual issues; Financial problems; and Need of information	Holistic tool covering a variety of domains.	There are gaps in the reliability testing administered, including test-retest reliability and inter-related reliability. There is also more research required to ascertain how implementation into the clinical setting should take place.	70.6% (12 out of 17).
*7. Missoula-VITAS Quality of Life Index* Schwartz et al. [49]	A tool aimed at assessing the quality of life of people with palliative and end of life needs. The tool was originally developed by Byock and Merriman [55], as a 25-item measure, which focused on how patients adapted to physical and functional deterioration. It was structured around five quality of life dimensions (symptom control, function, interpersonal issues, well-being, and transcendence).	The tool enabled opportunities to arise to discuss psychosocial and spiritual issues, which may not otherwise voluntarily emerge. The tool also enabled holistic, collaborative, person-centred care to materialise.	Improvements were also identified, including that organisational infrastructure support is required to ensure that the MVQOLI-R is used effectively, and that confidence would only improve with repeated use and possible training for staff. Moreover, as with the psychometric study, some items were found to be too complex for patients.	80.0%; 12 out of 15).
*8. Quality Care Questionnaire-End of Life* Yun et al. [27]	16-item measure that is relevant for patients with terminal illness. The questionnaire has undergone scrupulous development via a four-phase process, which has involved item generation and reduction, construction, pilot testing, and field testing	Good psychometric testing standards Reliability and validity testing strengthens the conviction that QLQ-EOL is appropriate for use with patients who have terminal cancer.	Cross-cultural studies may be needed to ascertain whether the QLQ-EOL is relevant for patients from other countries and cultures However, the authors also highlight that findings may be biased, and restricted in terms of generalisability.	(73.3%; 11 out of 15)

#### Collating summarising and reporting the results

This next section elaborates on the information provided by the charting process and provides a summary of the strengths and limitations of each PROM – please see Table 6 for a detailed account of the measures.

### Relevance to dignity

Five PROMs had explicit dignity foci, as evident in the title of the tool, dignity being directly mentioned in items, and/or dignity being addressed and described in the developmental process. These were the Palliative Nursing Quality Measure [40]; the Patient Dignity Inventory [41] the Jacelon Attributed Dignity Scale [42]; the MIDAM-LTC [43]; and the QCQ-EOL [27]. These PROMs were specifically developed to take into account the need to preserve dignity-conserving care [27]; acknowledge the broad landscape of distress-related concerns of people nearing end of life, and address the physical, psychosocial, existential, and spiritual aspects of the patient’s experience accepted that dignity was an integral theme within palliative care, and that commitment to this is a vital quality of palliative care nurses that enables them to respect humanity [40]; and appreciated the complex and unique nature of dignity [42, 43]. Two PROMs honed in on two particular types of dignity, “attributed dignity” (behaviour with respect to self and to others) [42] and “personal dignity” (individualistic, related to personal circumstances) [43, 44], thus offering a nuanced approach to measuring dignity.

The three other PROMs targeted dignity-related themes, such as kindness, humanity, respect [26]) compassion and person centred care [45]. The short version Problems and Needs in Palliative Care questionnaire [46] contains items that explore the impact of fear and difficulties upon the person’s wellbeing, the extent to which these are problematic, as well as allowing the person to indicate whether they wish for the healthcare professional to attend to this. The term dignity is not mentioned in the development of the questionnaire and does not appear in any items, but it was designed to be patient-centred [45]. Likewise, although The Quality of Communication Questionnaire [47] does not directly reference dignity, communication has been cited as main factor of dignity [48], so the tool can be regarded as relevant for assessing dignity. The Missoula-VITAS Quality of Life Index [49] includes a number of items under five domains, to assess the impact of aspects such as interpersonal and wellbeing factors on quality of life. However, since dignity has been recognised as a potential indicator of quality of life [50], it might have been helpful for the tool to more explicitly include this phenomenon.

### Strengths and limitations

The scoping review has successfully identified eight PROMs currently being used in palliative and/or end of life settings to measure dignity amongst people receiving care. The critical appraisal process highlighted some shortcomings in our critical appraisal tool, namely that not all psychometric criteria were covered, for example, ecological validity and factor analysis. This meant that if a study had subjected measures to those types of testing, this could not be acknowledged by our scoring. In addition, the Missoula-VITAS Quality of Life Index- Revised achieved a high score despite the authors suggesting that it was not suitable for use as a research outcome measure [49]. Moreover, the critical appraisal tool did not differentiate between the types of participants used during face validity testing. Therefore claims by researchers to have addressed face validity should be looked at more critically, since this validation is not always attained from the perceptions and responses of patient groups i.e. the end-users; rather it may be that the tool was tested amongst healthcare providers. This may result in a measure consisting of items that do not reflect areas that matter to patients, despite having undergone some form of face validity. However, the critical appraisal tool only partly informed the overall critical appraisal, and the description of measures allowed strengths and limitations to be considered beyond the tool’s criteria.

#### Implications for research and practice

The eight PROMs identified in this scoping have all been used in and are appropriate for palliative and/ or end of life research. The ones that scored highest on the critical appraisal tool could successfully be used in research studies. Although, some PROMs scored lower, other aspects of the PROMs (beyond the elements covered by the tool) were assessed, such as relevance for measuring dignity-related aspects. In this respect the PROMs performed well, which is why we make the claim that they are all apt for palliative and/or end of life care, since as stated earlier, levels of dignity should be measured since its values are prioritised by recipients of palliative and end of life care. Morever, all the PROMs addressed dignity-related themes, five were recognised as promoting an explicit agenda to assess dignity. All the measures included in the review were appraised to be clinically responsive, administrative manageable, had undergone some element of psychometric testing, and most demonstrated clearly that they could be administered at a baseline and subsequent time points. It would be advisable for further work to be conducted to test for effectiveness in the clinical context, before any of the measures are directly implemented clinically to measure dignity-conserving care. This would enable further development and evaluation of the PROMs to take place and would address some of the limitations that were identified. In particular, face validity should be performed with appropriate participants who represent the intended end-users of the tool.

## Conclusions

The review has identified eight PROMs which are designed to measure, at least in part, dignity amongst people with palliative and end of life care needs. All had undergone some level of psychometric testing. For the purposes of this review, a thorough critical appraisal procedure was applied to assess the PROMs in terms of strengths and limitations. Findings suggest that these eight PROMs could be used to help assess patients’ perceptions of dignity in palliative and/or end of life settings. Furthermore, they may also be appropriate for use in palliative and/or end of life intervention studies to capture significant changes in perceived dignity. However, researchers and clinicians should also consider the limitations of these measures, as highlighted in this scoping review, when making decisions around whether to implement them for research and clinical purposes.

### Recommendations

Based on the psychometric qualities and critical appraisal the outcome measure of choice from our review is the Patient Dignity Inventory developed by Chochinov et al. [41] which scored 14/15 and the *Missoula-VITAS Quality of Life Index* Schwartz et al. [49] which scored 12/15. We recommend researchers and clinicians use the measures with the highest scores. We recommend further validity and reliability testing and clinical research for the potentially useful other measures which scored lower regarding psychometric properties and in our critical appraisal. Findings of this review have useful implications for future research and practice. We have identified measures that can be used to measure dignity issues at the end of life. We recommend further PROM developed to improve person centred care at the end of life.
